# Double arterial cannulation strategy for acute type A aortic dissection repair: A 10-year single-institution experience

**DOI:** 10.1371/journal.pone.0211900

**Published:** 2019-02-06

**Authors:** Chun-Yu Lin, Chi-Nan Tseng, Hsiu-An Lee, Heng-Tsan Ho, Feng-Chun Tsai

**Affiliations:** 1 Chang Gung University, College of Medicine, Taoyuan, Taiwan; 2 Department of Cardiothoracic and Vascular Surgery, Chang Gung Memorial Hospital, Linkou, Taiwan; IRCCS Policlinico S. Donato, ITALY

## Abstract

**Background:**

Repair of acute type A aortic dissection (ATAAD) is a complex and emergent cardiovascular surgery that is associated with high perioperative morbidity and mortality. Each cannulation strategy has different benefits and drawbacks during cardiopulmonary bypass. Using a retrospective study design, we aimed to clarify the safety and efficacy of right axillary artery cannulation in combination with femoral artery cannulation compared to single arterial cannulation for ATAAD repair.

**Methods:**

From January 2007 to July 2017, 476 adult patients underwent ATAAD repair at a single institution. Patients were classified into groups according to their cannulation strategy: the double arterial cannulation (DAC) group (n = 377; 79.2%) or single arterial cannulation (SAC) group (n = 99; 20.8%). Preoperative demographics, surgical information, and postoperative recovery were compared between both groups. Survival and freedom from reoperation rates were analyzed using the Kaplan-Meier actuarial method.

**Results:**

Demographics, comorbidities, and surgical procedures were generally homogenous between the two groups, except for sex, age, and rate of extensive aortic repair. Patients who underwent DAC had lower in-hospital mortality (13.5% vs. 25.3%; *P* = 0.005) and lower incidence of malperfusion-related complications (18.8% vs. 30.3%; *P* = 0.011) than those who underwent SAC. During multivariate analysis, SAC was identified as an in-hospital mortality predictor (odds ratio, 2.81; 95% confidence interval, 1.52–5.17; *P* = 0.001), as were preoperative ventilator support, intraoperative extracorporeal membrane oxygenation installation, and postoperative malperfusion-related complications. Three-year cumulative survival and freedom from reoperation rates were 74.8% and 85.3% for the DAC group and 62.6% and 81.1% for the SAC group, respectively (*P* = 0.010 and 0.430, respectively).

**Conclusions:**

With acceptable short- and mid-term outcomes, DAC is effective and safe for establishing cardiopulmonary bypass during ATAAD repair.

## Introduction

**Fig 1:**
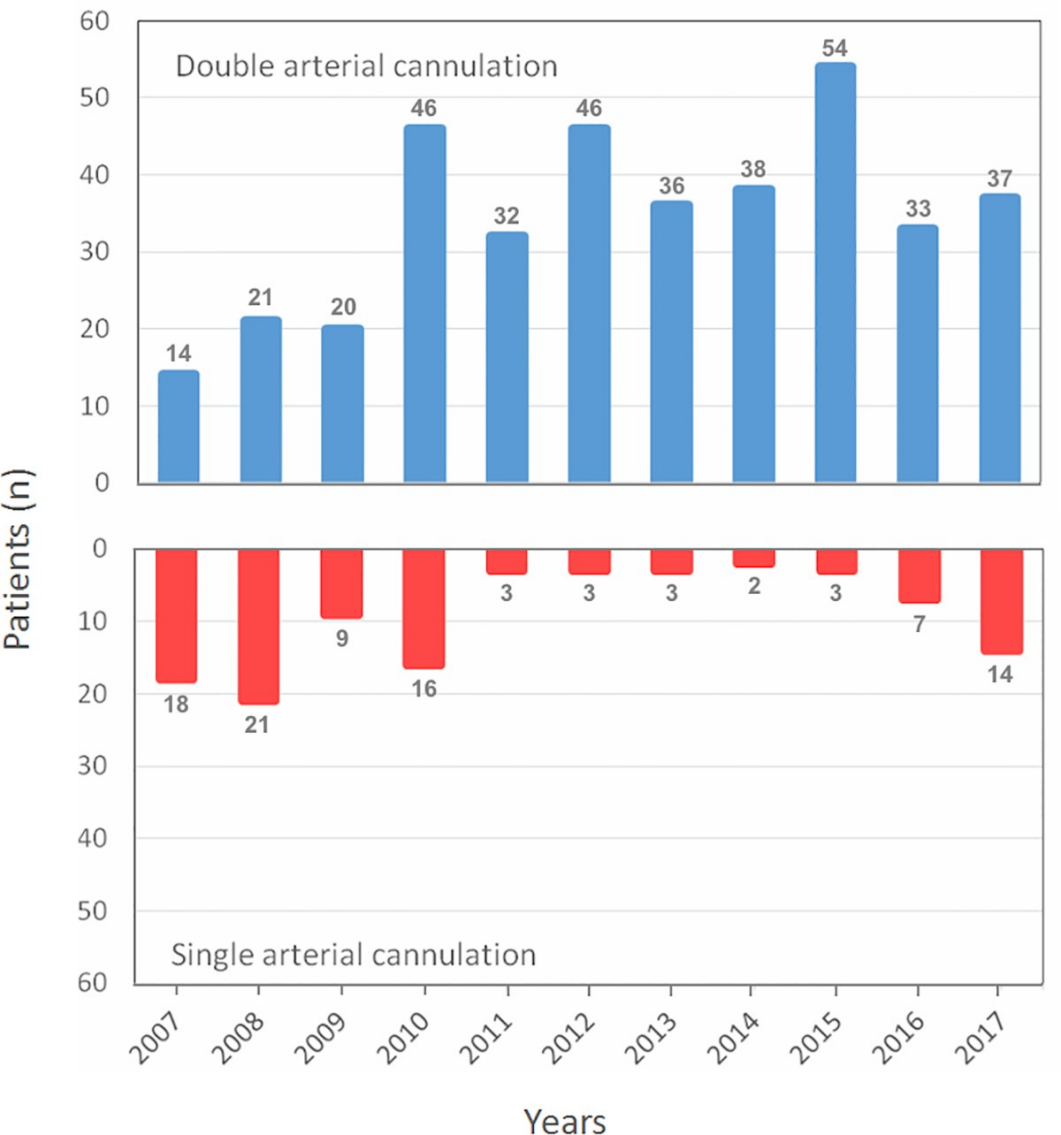
Distribution of cannulation strategies during the study period.

Acute type A aortic dissection (ATAAD) repair is a complex emergency cardiovascular procedure associated with high morbidity and mortality rates. Despite advancements in diagnostic tools, management algorithms, and surgical techniques in the past decades, ATAAD remains challenging for cardiothoracic surgeons. The in-hospital mortality rates ranged from 18% to 25% according to the international registry of acute aortic dissection, and have been reported as 17% by the German registry for acute aortic dissection type A [1, 2]. It is vital to use fast and safe arterial inflow to establish effective cardiopulmonary bypass (CPB) and avoid malperfusion in patients undergoing surgery for ATAAD. However, depending on the extent of dissection, surgical experience, and individual vascular anatomy, accomplishing this can be challenging. The most commonly used arterial cannulation sites are the right axillary and common femoral arteries. However, both of these access vessels provide different benefits and drawbacks during CPB, and there is still considerable debate regarding the optimal cannulation site for maximizing survival [3–6]. Furthermore, the results of using right axillary artery cannulation in combination with femoral artery cannulation are under-reported, with limited study cases [7, 8]. The technique of double arterial cannulation (DAC), as illustrated in Fig 1, was introduced at our institution in 2007. In 2010, it became the standard strategy for treating patients who undergo ATAAD repair at our institution. This study aimed to compare the early- and mid-term outcomes of DAC with those of single arterial cannulation (SAC), based on a retrospective analysis of the experiences of an individual center.

## Material and methods

### Patient enrollment and preoperative management

The present study was conducted with the approval of Chang-Gung medical foundation institutional ethics committee (No. 201800016B0). The need for informed consent was waived due to the retrospective nature of this study. After excluding those who were classified as having chronic aortic dissection, a total of 476 consecutive patients underwent emergency open ATAAD repair at a single institution between January 2007 and July 2017. All patients were diagnosed via helical computed tomography to confirm ATAAD in the emergency department. Before transferring the patient to the operating room (OR), we stabilized the patients’ hemodynamics with intravenous beta-blockers to maintain systolic blood pressure at <120 mmHg and heart rate at <60 bpm, in accordance with the 2010 American College of Cardiology/American Heart Association guidelines for thoracic aortic disease [9]. When patients presented to the emergency department with shock, we performed fluid resuscitation and on-site echocardiography. If cardiac tamponade was detected, pericardial fluid drainage was performed via subxiphoid pericardiotomy or echo-guided pericardiocentesis before patients were transferred to the OR. All procedures were performed on an emergency basis.

### Surgical management

After endotracheal intubation, arterial pressure catheters were inserted into both radial arteries. Transesophageal echocardiography was performed to evaluate cardiac function and severity of aortic regurgitation (AR). In general, the non-dissected right axillary artery and femoral artery were exposed and cannulated by two individual surgeons simultaneously before midline sternotomy and systemic heparinization. An 8-mm ring-reinforced polytetrafluoroethylene graft (Gore-Tex; W.L. Gore & Associates Inc., Flagstaff, AZ, USA) was routinely used to connect the arterial access and CPB circuits. The arterial accesses were then tested. When both access points were able to achieve a stable inflow rate of > 2.2–2.4 L/min/m^2^ with pressure gradient < 100 mmHg, we proceeded with the DAC strategy [10]. Otherwise, we converted the procedure to SAC by using the access point with better inflow. Following sternotomy, the right atrium was cannulated and CPB with deep hypothermia was initiated. The innominate artery, left common carotid artery, and left subclavian artery were exposed and looped with tapes. Once ventricular fibrillation occurred, the ascending aorta (AsAo) was cross-clamped, and the left ventricle was vented through the right superior pulmonary vein. Cardiac arrest was induced via a single dose of histidine-tryptophan-ketoglutarate solution (Custodiol; Essential Pharmaceuticals, LLC, Newtown, PA, USA) through the coronary orifice of the aortic root, or intermittent retrograde cold-blood cardioplegic solution through the coronary sinus. The dissected aorta was replaced with a Dacron prosthetic graft (Vascutek Gelseal; Terumo Cardiovascular Systems, Ann Arbor, MI, USA) according to the location of the entry tear and preoperative presentation. For most patients, the primary entry tear was resected if possible. However, in 27.3% of patients, the primary entry tear was left in place. The reasons include that a conservative surgical strategy, usually an isolated AsAo replacement for preventing its rupture was applied in critical patients with progressively unstable hemodynamic status refractory to resuscitation, the entry tear was repaired with compression stitches, or the entry tear was not found from preoperative images and intraoperatively. The AsAo was routinely replaced with aortic valve re-suspension. In most cases, proximal anastomosis was performed first. If the intimal tear extended to the aortic root with severe AR that was difficult to repair, aortic root replacement was then performed with a composite Valsalva graft. After proximal anastomosis was completed, the femoral arterial flow was temporarily suspended and the AsAo was de-clamped. If possible, the intima tear originating from the aortic arch was also resected and replaced. Otherwise, the AsAo was trimmed proximal to the innominate artery. Open distal anastomosis was performed under deep hypothermic circulatory arrest (18–22°C) and selective antegrade cerebral perfusion through the right axillary artery. The perfusion flow rate was set at 10–15 mL/kg/min, and the right radial arterial pressure was maintained at > 50 mmHg. For patients with an entry tear located at the distal arch and proximal descending aorta combined with preoperative malperfusion or thoracic aortic aneurysmal dilatation, a concomitant frozen elephant trunk procedure was performed. Once the distal anastomosis was complete, whole-body perfusion in the DAC group was resumed via the previously suspended flow of femoral artery and initiating systemic rewarming. In the SAC group, systemic perfusion was resumed via the solitary arterial access except the patients underwent axillary artery cannulation along with aortic arch replacement. Regarding this scenario, an additional central cannulation was performed at the Dacron graft of neo-aortic arch. Proximal and distal prosthetic grafts were connected with 4–0 polyproline running stitches. Before weaning from CPB, final confirmations including hemostasis, proper orientation of the prosthetic graft, and good competency of the aortic valve were performed.

### Data collection and statistical analysis

Statistical analyses were performed using SPSS for Windows (version 22.0; SPSS, Chicago, IL, USA). Data are presented as means ± standard deviations for continuous variables and as numbers/percentages for categorical data. For all analyses, statistical significance was set at *P* < 0.05. Univariate analyses were performed using the independent *t*, Mann–Whitney U, chi-squared, or Fisher’s exact tests to determine group differences in clinical demographics, surgical information, and postoperative complications. Significant variables in univariate analyses for in-hospital mortality (*P* < 0.05) were dichotomized based on cut-off values determined by receiver operating characteristic curve analyses. These dichotomized risk factors were tested in a prediction model of in-hospital mortality using a multivariate logistic regression analysis and the Hosmer-Lemeshow test [11]. The Kaplan-Meier method was used to construct the 3-year cumulative survival and 3-year freedom from reoperation rates, which were compared using the log-rank test. The Penn classification, a mortality risk-stratification system by ischemic presentation was used to analyze the outcomes of DAC and SAC groups [12].

## Results

### Patient demographics

The clinical demographics, comorbidities, preoperative conditions, and clinical presentations for the DAC and SAC groups are shown in Table 1. The DAC group was younger with less female patients compared to the SAC group. Hypertension was the most prevalent comorbidity, accounting for > 70% of cases in both groups. The average interval from the emergency department to the OR was 5.5 ± 1.5 hours. A total of 89 (18.7%) of patients were diagnosed with intramural hematoma. Intractable pain was the most prevalent clinical presentation (333, 70.0%), followed by hemopericardium (133, 27.9%;) and malperfusion (72, 15.1%). No disparity in clinical presentations was found between the groups.

**Table 1:** Preoperative patient characteristics according to the patient group.

Parameters	Overall	DAC	SAC	*P* value
	n = 476	n = 377	n = 99	
Clinical demographics				
Sex (female, n, %)	136, 28.6	98, 26.0	38, 38.4	.012
Age (years)	56.0 ± 14.1	55.3 ± 13.7	58.7 ± 15.3	.030
Body mass index (kg/m^2^)	26.1 ± 4.8	26.2 ± 5.2	26.0 ± 3.2	.562
Comorbidities				
Diabetes mellitus (n, %)	29, 6.1	20, 5.3	9, 9.1	.124
Hypertension (n, %)	341, 71.6	271, 71.9	70, 70.7	.454
Creatinine (mg/dL)	1.5 ± 1.4	1.5 ± 1.5	1.4 ± 0.4	.591
ESRD (n, %)	9, 1.9	9, 2.4	0, 0	.120
Preoperative condition				
SBP (mmHg)	95.8 ± 14.1	96.4 ± 13.5	93.6 ± 16.2	.075
SBP < 90 mmHg (n, %)	93, 19.5	68, 18.0	25, 25.3	.073
LVEF (n, %)	62.8 ± 9.7	63.2 ± 9.5	61.7 ± 10.4	.176
LVEF < 50% (n, %)	79, 16.6	58, 15.4	21, 21.2	.110
Ventilator support (n, %)	20, 4.2	17, 4.5	3, 3.0	.373
Time from ED to OR (hr)	5.5 ± 1.5	5.6 ± 1.5	5.4 ± 1.6	.190
Clinical presentation				
DeBakey type II (n, %)	55, 11.6	39, 10.3	16, 16.2	.079
Intramural hematoma (n, %)	89, 18.7	69, 18.3	20, 20.2	.381
Intractable pain (n, %)	333, 70.0	263, 69.8	70, 70.7	.480
AR with heart failure (n, %)	70, 14.7	54, 14.3	16, 16.2	.375
Hemopericardium (n, %)	133, 27.9	101, 26.8	32, 32.3	.167
Malperfusion (n, %)	72, 15.1	58, 15.4	14, 14.1	.449
Limb ischemia (n, %)	36, 7.6	31, 8.2	5, 5.1	.288
Brain stroke (n, %)	15, 3.2	10, 2.7	5, 5.1	.224
Paraplegia (n, %)	8, 1.7	8, 2.1	0. 0	.144
Visceral ischemia (n, %)	5, 1.1	3, 0.8	2, 2.0	.288
AMI (n, %)	8, 1.7	6, 1.6	2, 2.0	.521
Penn classification				
No ischemia (n, %)	330, 69.3	267, 70.8	63, 63.6	.168
Localized ischemia (n, %)	53, 11.1	42, 11.1	11, 11.1	.993
Generalized ischemia (n, %)	74, 15.5	52, 13.8	22, 22.2	.039
Combined ischemia (n, %)	19, 4.0	16, 4.2	3, 3.0	.583

AMI, acute myocardial infarction; AR, aortic regurgitation; DAC, double arterial cannulation; ED, emergency department; ESRD, end-stage renal disease; LVEF, left ventricular ejection fraction; OR, operating room; SAC, single arterial cannulation; SBP, systolic blood pressure

### Surgical information

Table 2 provides detailed information regarding surgical variables. In the SAC group, 82 (82.8%), 16 (16.2%), and 1 (1.0%) of patients underwent femoral artery, axillary artery, and direct AsAo cannulation, respectively. The technical success rate of DAC was 98.2% (377/384) in the present study. Only seven patients shifted to SAC strategy due to failure of DAC strategy. In terms of aortic repair procedures, more extensive distal repairs were performed for the DAC group, including arch replacement and the frozen elephant trunk procedure. The prevalence of primary entry tear exclusion were similar, accounting for > 70% of cases in both groups. For 130 patients without excluding the primary entry tear, 44 (33.8%) were located at the aortic arch, 24 (18.5%) at the descending aorta, 6 (4.6%) at the aortic root, and 56 (43.1%) were not found to have a definite intima tear. The time spans of CPB, aortic cross-clamping, and circulatory arrest were not significantly different between both groups. For 82 patients underwent femoral artery cannulation in the SAC group, 4 applied antegrade cerebral perfusion strategy by using balloon-tip perfusion catheters, and 78 applied retrograde cerebral perfusion strategy through superior vena cava. Moreover, patients with DAC had higher body temperatures during circulatory arrest. A total of 52 (10.9%) patients required Kerlix packing with delayed sternum closure, and 13 (2.7%) underwent extracorporeal membrane oxygenation (ECMO) installation in the OR due to intraoperative myocardial failure.

**Table 2:** Surgical information according to patient group.

Parameters	Overall	DAC	SAC	*P* value
	n = 476	n = 377	n = 99	
Femoral artery cannulation (n, %)		377, 100	82, 82.8	
Axillary artery cannulation (n, %)		377, 100	16, 16.2	
AsAo cannulation (n, %)		0, 0	1, 1.0	
Aortic repair procedures				
Entry tear exclusion (n, %)	346, 72.7	273, 72.4	73, 73.7	.450
Root replacement (n, %)	54, 11.3	43, 11.4	11, 11.1	.549
Isolated AsAo replacement (n, %)	315, 66.2	240, 63.7	75, 75.8	.015
Arch replacement (n, %)	117, 24.6	103, 27.3	14, 14.1	.004
Frozen elephant trunk (n, %)	38, 8.0	37, 9.8	1, 1.0	.001
Cardiopulmonary bypass time (min)	263.8 ± 78.5	261.3 ± 75.4	273.4 ± 89.2	.171
Aortic clamping time (min)	167.4 ± 56.6	167.2 ± 55.0	168.1 ± 62.7	.884
Circulatory arrest time (min)	50.9 ± 26.9	51.2 ± 27.8	49.8 ± 23.2	.658
Hypothermia temperature (°C)	19.9 ± 2.1	20.1 ± 2.1	19.0 ± 2.1	.001
Delayed sternum closure^a^ (n, %)	52, 10.9	43, 11.4	9, 9.1	.325
ECMO support (n, %)	13, 2.7	11, 2.9	2, 2.0	.471

^a^Kerlix packing for uncontrolled coagulopathy and planned secondary exploration.

AsAo, ascending aorta; DAC, double arterial cannulation; ECMO, extracorporeal membrane oxygenation; SAC, single arterial cannulation

### Postoperative recovery and morbidities

As Table 3 illustrates, patients with DAC had significantly lower in-hospital mortality rates than patients who underwent SAC. In addition, occurrence of postoperative malperfusion-related complications was more prevalent in patients who underwent SAC. Patients with SAC also had a high incidence of other complications, including visceral ischemia, limb ischemia, deep sternal wound infection, prolonged mechanical ventilation, and ICU readmission. However, these differences were not statistically significant. The subgroup analyses of outcomes for patients having high risks with hospital mortality and postoperative complications are illustrated in Table 4 [12–15], which shows that DAC provided significantly lower postoperative malperfusion-related complications among the subgroups with preoperative malperfusion, the Penn classification-localized ischemia, and body mass index > 25, and compared to those who underwent SAC. In patients older than 70 years, the DAC group demonstrated trends of better survival and lower malperfusion-related complications. In patients who used axillary artery for cannulation access, the hospital mortality and postoperative malperfusion-related complication rates were 13.5% (51/377) and 18.8% (71/377) for the DAC group and 25.0% (4/16) and 31.3% (5/16) for the SAC group, respectively (*P* = 0.195 and 0.218, respectively).

**Table 3:** Postoperative mortality and morbidity according to the patient group.

Parameters	Overall	DAC	SAC	*P* value
	n = 476	n = 377	n = 99	
Hospital mortality (n, %)	76, 16.0	51, 13.5	25, 25.3	.005
Bleeding (n, %)	14, 2.9	9, 2.4	5, 5.1	.163
Brain stem failure (n, %)	10, 2.1	6, 1.6	4, 4.0	.131
Myocardial failure (n, %)	33, 6.9	24, 6.4	9, 9.1	.342
Sepsis (visceral ischemia-related, n, %)	8, 1.7	5, 1.3	3, 3.0	.240
Sepsis (non-visceral ischemia-related, n, %)	11, 2.3	7, 1.9	4, 4.0	.198
Hemodialysis (n, %)	43, 9.0	34, 9.0	9, 9.1	.557
Transfusion at 24 h after surgery				
RBC^a^ (units)	8.1 ± 6.7	8.2 ± 6.7	7.9 ± 7.0	.743
Plasma^b^ (units)	7.3 ± 6.2	7.3 ± 6.1	7.3 ± 6.6	.930
Platelet (units)	17.4 ± 11.8	17.5 ± 11.7	17.3 ± 12.2	.882
Reoperation for bleeding (n, %)	73, 15.3	57, 15.1	16, 16.2	.452
Atrial fibrillation (n, %)	28, 5.9	24, 6.4	4, 4.0	.272
Brain stroke (n, %)	64, 13.4	45, 11.9	19, 19.2	.046
Infarction (n, %)	56, 11.8	38, 10.1	18, 18.2	.024
Hemorrhage (n, %)	8, 1.7	7, 1.9	1, 1.0	.479
Delirium (n, %)	94, 19.7	76, 20.2	18, 18.2	.389
Seizure (n, %)	26, 5.5	24, 6.4	2, 2.0	.065
Visceral ischemia (n, %)	11, 2.3	7, 1.9	4, 4.0	.177
Limb ischemia (n, %)	16, 3.4	11, 2.9	5, 5.1	.223
Malperfusion-related complications^c^ (n, %)	101, 21.2	71, 18.8	30, 30.3	.011
Pneumonia (n, %)	47, 9.9	39, 10.3	8, 8.1	.323
Deep sternal wound infection (n, %)	13, 2.7	9, 2.4	4, 4.0	.276
Extubation time (h)	98.9 ± 297.4	93.8 ± 313.6	120.9 ± 214.0	.463
Ventilator support > 72 h (n, %)	139, 29.2	109, 28.9	30, 30.3	.438
Tracheostomy (n, %)	16, 3.4	13, 3.4	3, 3.0	.566
ICU stay (days)	7.7 ± 16.0	7.7 ± 17.3	7.7 ± 9.4	.989
ICU readmission (n, %)	27, 5.7	19, 5.0	8, 8.1	.177
Hospital stay (days)	26.7 ± 52.0	26.7 ± 56.0	26.4 ± 32.6	.951

^a^Red blood cell transfusion including the amount of whole blood and packed red cell concentrate.

^b^Plasma transfusion including the amount of fresh-frozen plasma and cryoprecipitate.

^c^Occurrence of renal failure, brain infarction, visceral ischemia, and limb ischemia.

DAC, double arterial cannulation; ICU, intensive care unit; SAC, single arterial cannulation

**Table 4:** Subgroup analyses of outcomes for high-risk patients.

Parameters	Overall	DAC	SAC	*P* value
Preoperative malperfusion (n)	72	58	14	
Hospital mortality (n, %)	9, 12.5	7, 12.1	2, 14.3	.560
Malperfusion-related complications (n, %)	14, 19.4	8, 13.8	6, 42.9	.023
Penn classification (localized ischemia, n)	53	42	11	
Hospital mortality (n, %)	6, 11.3	4, 9.5	2, 18.2	.420
Malperfusion-related complications (n, %)	8, 15.1	3, 7.1	5, 45.5	.002
Penn classification (generalized ischemia, n)	74	52	22	
Hospital mortality (n, %)	16, 21.6	11, 21.2	5, 22.7	.881
Malperfusion-related complications (n, %)	16, 21.6	12, 23.1	4, 18.2	.640
Penn classification (combined ischemia, n)	19	16	3	
Hospital mortality (n, %)	3, 15.8	3, 18.8	0, 0	.414
Malperfusion-related complications (n, %)	6, 31.6	5, 31.3	1, 33.3	.943
Age > 70 years (n)	87	63	24	
Hospital mortality (n, %)	19, 21.8	12, 19.0	7, 29.2	.229
Malperfusion-related complications (n, %)	15, 17.2	10, 15.9	5, 20.8	.398
Body mass index >25 (n)	236	186	50	
Hospital mortality (n, %)	36, 15.3	23, 12.4	13, 26.0	.019
Malperfusion-related complications (n, %)	52, 22.0	36, 19.4	16, 32.0	.045

DAC, double arterial cannulation; SAC, single arterial cannulation

### Prognostic factors associated with in-hospital mortality

Table 5 provides the results of logistic regression analyses. Four significant prognostic factors for in-hospital mortality were identified: SAC, preoperative ventilator support, ECMO initiated in the OR, and postoperative malperfusion-related complications.

**Table 5:** Logistic regression results for hospital mortality.

Parameters	β-coefficient	Standard error	Odds ratio, 95% CI	*P* value
Univariate logistic regression				
SAC	0.770	0.276	2.16 (1.26–3.71)	.005
Preoperative ventilator support	1.776	0.467	5.91 (2.37–14.75)	< .001
Isolated AsAo replacement	- 0.683	0.254	0.51 (0.31–0.83)	.007
Arch replacement	0.712	0.266	2.04 (1.21–3.44)	.008
CPB time > 208 min^a^	1.416	0.478	4.12 (1.62–10.52)	.003
Aortic clamp time > 175 min^b^	0.722	0.253	2.06 (1.25–3.38)	.004
Circulatory arrest time > 62 min^c^	0.927	0.257	2.53 (1.53–4.19)	< .001
ECMO support	2.588	0.615	13.30 (3.98–44.42)	< .001
Hemodialysis	1.184	0.349	3.27 (1.65–6.47)	.001
Brain stroke	0.671	0.321	1.96 (1.04–3.67)	.036
Malperfusion-related complications	1.106	0.269	3.02 (1.79–5.12)	< .001
Multivariate logistic regression^d^				
SAC	1.031	0.321	2.81 (1.52–5.17)	.001
Preoperative ventilator support	2.041	0.504	7.70 (2.87–20.69)	< .001
ECMO support	2.641	0.646	14.03 (3.96–49.74)	< .001
Malperfusion-related complications	1.041	0.519	2.83 (1.03–7.83)	.045

^a^AUROC 0.697; sensitivity 93.4%; specificity 22.5%; Youden Index 0.159

^b^AUROC 0.601; sensitivity 56.6%; specificity 62.0%; Youden Index 0.186

^c^AUROC 0.595; sensitivity 46.1%; specificity 74.8%; Youden Index 0.209

^d^CPB time, aortic clamp time, and circulatory arrest time were not conducted to multivariate logistic regression analysis due to the poor sensitivity and specificity.

AsAo, ascending aorta; AUROC, area under the receiver operating characteristic curve; CI, confidence interval; CPB, cardiopulmonary bypass; ECMO, extracorporeal membrane oxygenation; SAC, single arterial cannulation

### Cumulative 3-year survival and freedom from reoperation rates

**Fig 2:**
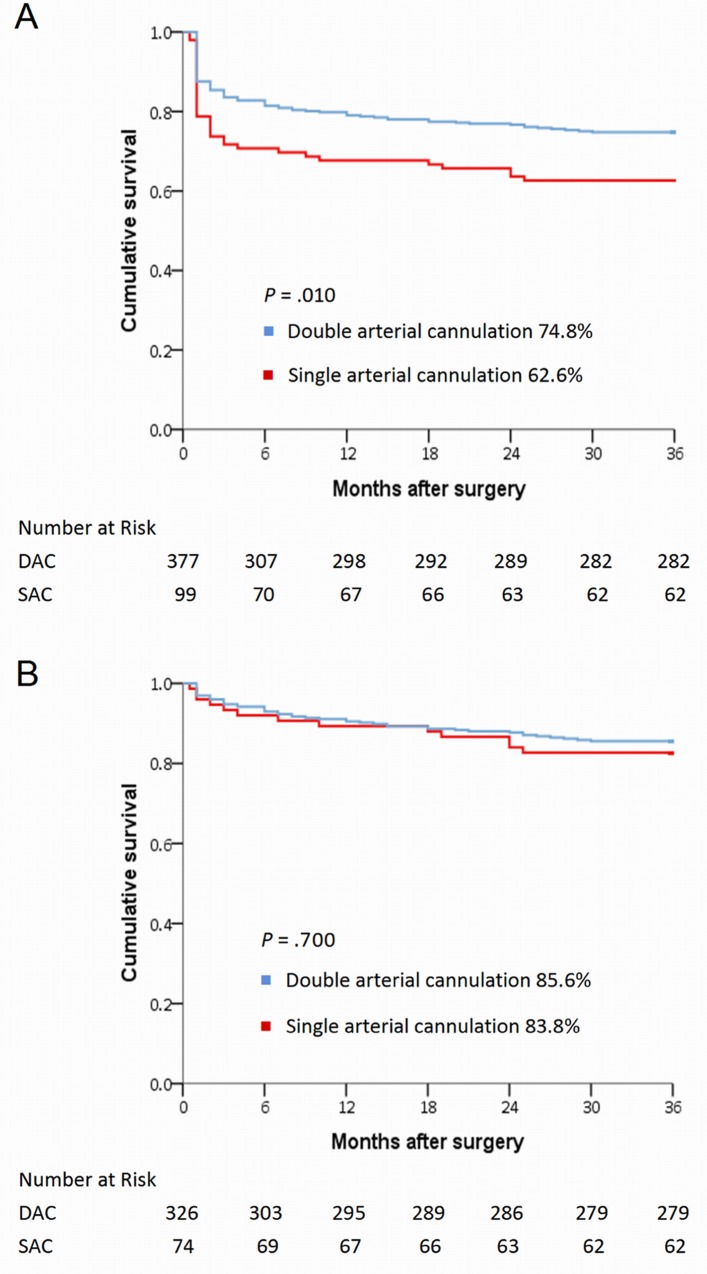
(A) Kaplan-Meier curves of 3-year cumulative survival for 476 patients; and (B) Kaplan-Meier curves of 3-year cumulative survival for 400 patients (excluding those with in-hospital mortality) stratified by cannulation strategies.

**Fig 3:**
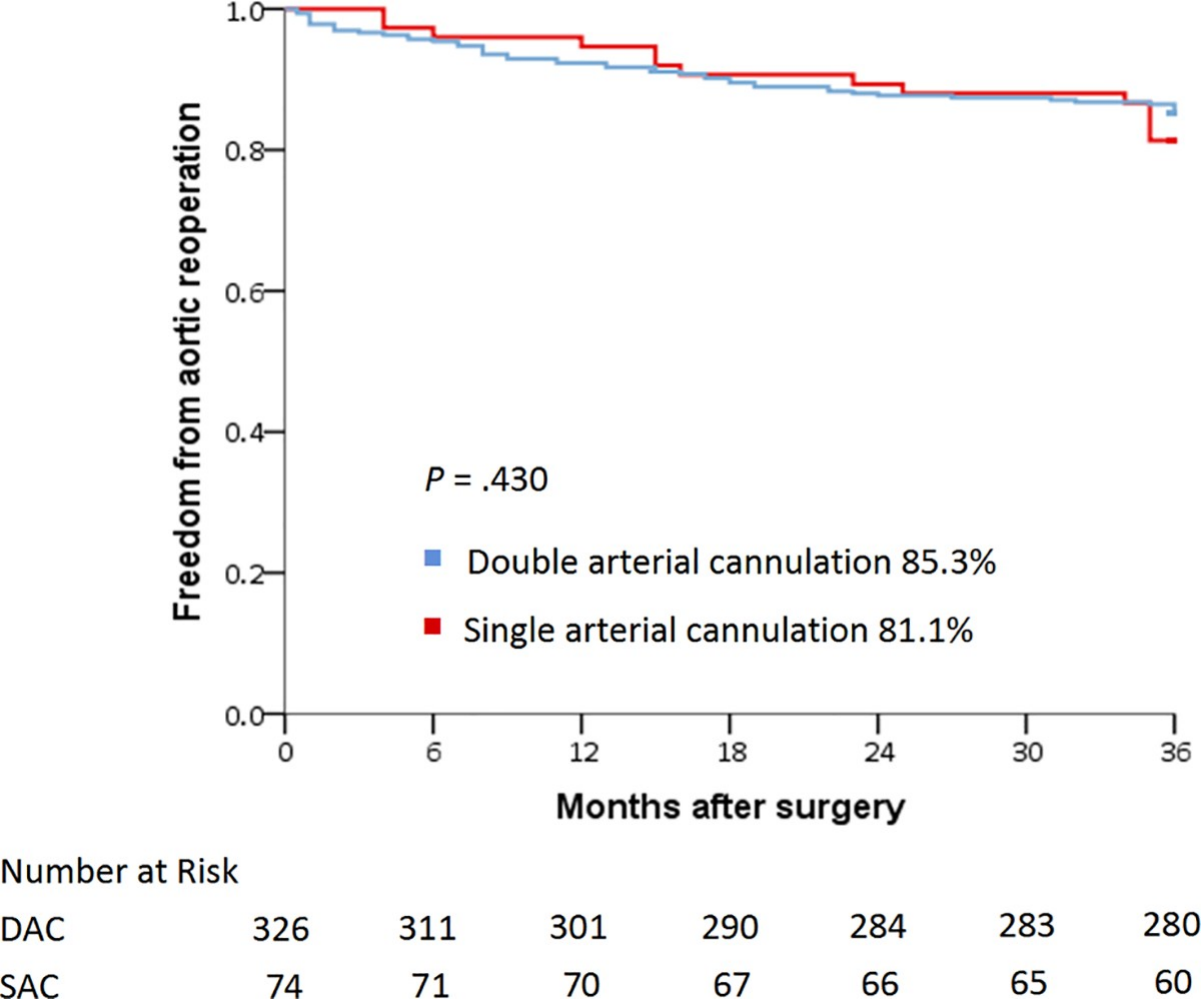
Kaplan-Meier curves of 3-year freedom from aortic reoperation stratified by cannulation strategies.

The 3-year cumulative survival rate was significantly higher for overall patients who underwent DAC than for those who underwent SAC (Fig 2A). However, there is no inter-group disparity of 3-year cumulative survival after excluding the in-hospital mortality (Fig 2B). Moreover, for patients who survived to discharge, the 3-year freedom from aortic reoperation rate was not significantly different between the two groups (Fig 3).

## Discussion

ATAAD is an emergency surgery associated with high mortality and morbidity. The use of a fast and safe arterial inflow for establishing effective CPB is crucial. An appropriate CPB maintains adequate systemic perfusion and prevents occurrence of end-organ malperfusion during surgery. Isolated femoral artery cannulation and axillary artery cannulation have been widely adopted and well-discussed in previous literatures [4, 6, 16, 17]. Although the use of DAC provides reliable circulatory support for the repair of ATAAD, results of this cannulation strategy are scarcely addressed in studies, with limited cases available [7, 8]. In the present study, 87% (326/377) of patients who underwent DAC survived; in addition, the cumulative survival rate at 3 years post-surgery was 75% (282/377). At an institution experienced with this modality, DAC may result in better satisfactory short- and mid-term outcomes than SAC.

### Differences between SAC and DAC

The femoral artery has been used as the primary cannulation site for CPB during cardiac surgery for more than 40 years [18]. As previous studies revealed, cannulation with the femoral artery for ATAAD repair may induce more injury in the dissected aorta and end-organ malperfusion resulting from true vascular lumen compression or retrograde thromboembolization [19, 20]. These malperfusion-related complications are associated with longer in-hospital durations and higher mortality rates [21, 22]. Since the late 1990s, the axillary artery has been used as the cannulation site for reoperations and cases involving aortic pathology, including ATAAD repair [23–25]. A growing trend of using axillary artery cannulation during the past two decades has been reported [17]. By providing antegrade cerebral perfusion under hypothermic circulatory arrest, better neurological outcomes have been achieved [22, 26]. However, there are still several drawbacks associated with solitary axillary artery cannulation, including limited flow rate, greater technical demand, and intraoperative dissection of the innominate artery [7, 27, 28]. Therefore, we attempted to perform antegrade and retrograde arterial flow simultaneously to achieve optimal systemic perfusion and avoid these fatal shortcomings. Furthermore, for patients who presented with an extensive vascular involvement of ATAAD, left axillary artery or ipsilateral femoral artery cannulation was considered as additional cannulation access if monitoring of peripheral arterial pressure and pump flow demonstrates abnormal signs.

The DAC strategy has been performed at our institution for 10 years. The time span from vascular exposure to completing ringed graft anastomosis and initiating CPB is usually less than 15 minutes. Therefore, we suspect that the higher technical demands of this procedure should not be a major concern at an experienced cardiovascular center familiar with this approach. Furthermore, by using DAC, the pump flow for CPB can be sustained more easily in a physiological range, even for patients in shock or with a small access vessel profile. Although cannulation with femoral artery was recognized as a risk factor for inferior outcome [19, 20], the prevalence of femoral arterial cannulation is 100% in the DAC group, higher than 82.8% in the SAC group. Therefore, we suspect that the use of femoral artery may not be the main factor inducing disparity of results between the DAC and SAC groups. In the present study, the DAC group had low mortality and malperfusion-related complication rates. We believe that it is safe and efficient to combine femoral artery cannulation with axillary cannulation to establish CPB in the context of ATAAD repair. Even for patients with higher surgical risks, such as preoperative malperfusion, old age, and obesity, this cannulation strategy provided acceptable outcomes. Furthermore, the mid-term follow-up also showed promising results.

The malperfusion observed in aortic dissection is highly correlated to its complex anatomic interactions between the true lumen and the false lumen along the entire dissected aorta. Furthermore, this anatomic interactions can be dynamic and even influenced by the surgical procedure itself. However, the intraoperative surveillance of malperfusion among visceral organs and lower extremities are usually inadequate during ATAAD repair. DAC may be more reliable then SAC to reach an adequate systemic perfusion by underwent antegrade and retrograde perfusion simultaneously, especially to the dissected branch-vessels containing re-entry tears and compromised true lumen. In other words, a bi-direction flow may have higher possibility to provide a stable whole-body perfusion then a single direction flow. In the present study, the benefit of reducing postoperative malperfusion-related complications in the subgroup of preoperative malperfusion is well addressed (13.8% vs. 42.9%; *P* = 0.023) at Table 4. However, further studies for clarifying the detail physiologic mechanism should be conducted in the future. For patients underwent DAC strategy, there were no conflict of pump flow being found intraoperatively in the present study, and this technique merits serious consideration in all patients underwent ATAAD repair. Furthermore, DAC revealed significantly lower postoperative malperfusion-related complications among the subgroup of Penn classification-localized ischemia. However, there were no similar benefits provided by DAC in the subgroups of generalized ischemia and combined ischemia. These findings gave us an important message: The DAC strategy should be considered as the preferable treatment choice for patients who presented with preoperative malperfusion but under stable hemodynamic status as its benefit on reducing postoperative malperfusion-related complications. However, probably due to a more extended process of vessels exposure, this advantage could be attenuated for patients with unstable hemodynamics.

### Cerebral protection during circulatory arrest

Cerebral perfusion via the right axillary artery may not provide sufficient perfusion to the left cerebral hemisphere in some patients with an embryologically dysplastic Circle of Willis, which connects the blood circulation between the bilateral cerebral hemispheres. As Merkkola *et al*. reported [29], this vascular malformation can cause hypo-perfusion of 14–17% to the contra-lateral brain when using solitary axillary artery cannulation during aortic surgery. Therefore, using transcranial cerebral oximetry is helpful to detect the insufficient cerebral perfusion during circulatory arrest. However this modality is not available in the institute. Furthermore, bilateral antegrade cerebral perfusion, which combines right axillary artery cannulation with a supplementary catheter in the left carotid artery to lessen neurological complications [30], was only applied on 14 patients in the present study. For the reasons described above and the prolonged averaged circulatory arrest time (50.9 ± 26.9 min), the deep hypothermic circulatory arrest strategy at 18–22°C was used to reduce undetected intraoperative cerebral malperfusion. Furthermore, the SAC group demonstrated a lower body temperatures during circulatory arrest, which may be related with the commonly adopted retrograde cerebral perfusion strategy, and a more primitive concept for cerebral protection before 2010 in this cohort.

### Three-year outcomes

DAC yielded acceptable in-hospital and 3-year survival rates. Furthermore, even with more extensive distal repair in the DAC group, there were no intergroup differences in 3-year freedom from aortic reoperation. Due to the comparable proportions of primary entry tear exclusions in the two groups, the incidence of reoperation should be similar, regardless of the influence of cannulation strategy. As Concistrè et al. [31] reported, reoperation after ATAAD repair is mainly associated with a patent false lumen, tissue glue use, and aortic root preservation during the initial surgery, rather than the cannulation technique. However, a 3-year follow-up may be too short to stratify an inter-group disparity of freedom from aortic reoperation in the present study. A extended follow-up interval is necessary to clarify whether the more prevalent arch replacements and frozen elephant trunk procedures in the DAC group could lead to a favorable outcome of the distal aorta.

### Limitations

This study used a retrospective and non-randomized control design. Therefore, bias of selecting patients might have influenced the homogeneity of the groups, including the surgical time, cerebral protection strategies, and observed disparity of gender and age. However, female and advanced age may not result in adverse outcomes according to previous studies [32–34]. Furthermore, the repair procedure was decided by individual physicians, and differences in the extent of aortic replacement and secondary intervention strategies might have affected the final outcomes. During the 10-year period, CPB technology, myocardial protection strategies, and ICU care protocols may have varied. Finally, as a retrospective study, some hemodynamic data, laboratory profiles, and inotropic medication dosage information may have been incomplete, thus hindering more detailed analyses of physiological fluctuations during the perioperative course. The detail physiologic mechanism for providing benefits of DAC strategy is not entirely understood in the present study. Further studies for clarifying this issue should be conducted in the future.

## Conclusions

By providing reliable arterial flow and stabilizing systemic tissue perfusion during surgery, using DAC to establish CPB can be a safe strategy during ATAAD repair. When performed at an experienced institution, DAC may yield satisfactory short- and mid-term outcomes.
